# Strangury from Blisters

**Published:** 1854-01

**Authors:** 


*Strangury from Blisters.*—Dr. Anderson, of Alabama, believes that strangury can uniformly be prevented "by smearing the plaster with oil of turpentine" before applying it.—*From the N. H. Journal of Medicine.*

